# Estimating transmission intensity from *P.falciparum* serological data using antibody density models

**DOI:** 10.1186/1475-2875-11-S1-P79

**Published:** 2012-10-15

**Authors:** Emilie Pothin, Neil Ferguson, Chris Drakeley, Azra Ghani

**Affiliations:** 1 MRC Centre for Outbreak Analysis & Modelling, Department of Infectious Disease Epidemiology, Imperial College London, London, UK; 2 Department of Immunology, London School of Hygiene & Tropical Medicine, London, UK

## Background

Serological data are increasingly being used to monitor malaria transmission intensity and have been demonstrated to be particularly useful in areas of low transmission where traditional measures such as EIR and parasite prevalence are limited. The seroconversion rate is usually estimated using catalytic models in which the measured antibody levels are used to categorise individuals as seropositive or seronegative. One limitation of this approach is that the cut-off between positive and negative is arbitrary. Furthermore, the continuous variation in antibody levels is ignored thereby potentially reducing the precision of the estimate.

## Material and methods

To overcome these limitations we developed a series of age-specific density models which mimic antibody acquisition and loss. These were fitted to antibody titre data from 12 villages at different altitude in Northern Tanzania to estimate the rate of acquisition of antibodies as a measure of transmission intensity for multiple *P.falciparum* endemic settings.

## Results

Our results indicate that a model in which the boost in antibodies following exposure depends on the existing antibody level (with a decline in the size of the antibody boost with higher levels of circulating antibodies) and that includes variation between individuals in the size of the response fits the data well. We obtained a high correlation between our new estimates of the force of infection and estimates of the seroconversion rate obtained from the original catalytic model (r=0.95). Our estimates were also highly correlated with the estimated EIR (r=0.83) and parasite prevalence (r=0.67) in these 12 villages. The precision of the estimates obtained using the density model was greater than those obtained using the catalytic model.

## Conclusion

This approach, if validated across different epidemiological settings, could be a useful alternative model to estimate transmission intensity from serological data which avoids the need for an arbitrary cut-off value.

